# Current progress and future perspective of super-enhancers: a viable and effective bridge between the transcriptional apparatus and disease

**DOI:** 10.3389/fgene.2025.1611905

**Published:** 2025-07-02

**Authors:** Ran Wang, Aiying Li, Zongran Pang

**Affiliations:** ^1^ School of Pharmacy, Key Laboratory of Ethnomedicine of Ministry of Education, Minzu University of China, Beijing, China; ^2^ School of nursing, Hebei University of Chinese Medicine, Shijiazhuang, Hebei, China

**Keywords:** super-enhancer, cell fate, cancer, CNS system disease, autoimmune disease

## Abstract

Super-enhancers are a super-cluster of enhancers formed by serially arranged regulatory elements that can strongly drive the expression of cell-related genes. Hundreds of SEs in cells affect cell identity and fate-determining processes. Previous studies have verified that the expression of pathogenic genes is highly correlated with the abnormal activation of SEs in malignant tumorigenesis, dementia, diabetes, and many autoimmune diseases. Also, enhancer RNAs (eRNAs) can be regarded as crucial markers for SEs. Here, we summarize the discovery process and basic concepts of SEs, describe the structural characteristics and functional regulation of SEs in different tumor diseases, Alzheimer’s disease, and immune-related diseases, with a focus on typical diseases such as rheumatoid arthritis, systemic lupus erythematosus, and multiple sclerosis. In this review, we also discuss the potential clinical applications of SE, as well as the research prospects in this field.

## 1 Introduction

An enhancer is a DNA sequence in the genome that can regulate the expression of target genes spatially and precisely during cellular development and differentiation (Simakov et al., 2013). The ENCODE Project Consortium has speculated that there are approximately 400,000 putative enhancers in the human genome based on different regulatory elements of the genome, which can be classified according to seven different chromosome states (The ENCODE Project Consortium, 2012). In addition, as more cell types are analyzed and studied, the number of human enhancers has increased to more than one million (Jia et al., 2020; Levine et al., 2014).

**FIGURE 1:**
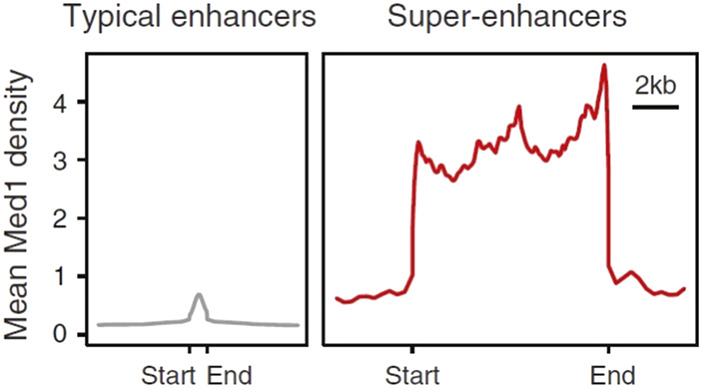
Comparison of signal strength between typical enhancers and SEs (Hnisz et al., 2013). Y-axis represents the overall signal value of SEs and enhancers.

Cells store genetic information in DNA, synthesize mRNA through transcription, and translate mRNA into proteins with specific biological functions. Since 1985, this process has been known as the “central dogma” (Moldave, 1985), confirming the transcription process is divided into multiple stages, including initiation, elongation and termination (Gayon, 2016). RNA polymerase II is regarded as the core factor regulating the process of gene transcription. Gene expression is mediated by common transcription factors, promoters, enhancers, mediators, cohesion, insulators, and silencers (Juven-Gershon and Kadonaga, 2010). Normally, common transcription factors can bind to the promoters of genes and thereby stimulate gene expression. However, unlike promoters, enhancers can regulate gene expression in a nondirectional manner, and the distance from target genes may be highly variable, they can be located upstream or downstream of genes, or within introns, or even within genes that have different chromatin profiles (Li et al., 2023; Lomvardas et al., 2006; Spilianakis et al., 2005). In some cases, a single enhancer can even regulate the expression of multiple genes (Plank and Dean, 2014). In cells, the activity of specific enhancers can be limited by a variety of factors and only elevates target gene expression within specific tissue and cell types, specific time points, or special physiological, pathological, and environmental conditions. Therefore, this dynamic regulation of enhancer activity has been shown to be critical in cellular differentiation (Ong and Corces, 2011). At that time, Richard A. Young’s lab proposed the concept of SEs on the basis of the mean density of Mediator coactivator (Med1) compared to the typical enhancers by using ChIP-Seq (Whyte et al., 2013). The information generated by ChIP-seq has greatly facilitated the understanding of the mechanisms by which enhancers, transcription factors, co-factors and histone modifications regulate gene expression. ChIP-seq fold the difference for enhancer features, such as Mediator, H3K27ac, H3K4me1, and DNaseI hypersensitivity between SEs vs. typical enhancers. SE region span is typically 8 to 20 Kb, which is much higher than typical enhancer of 200–300 bp region span. In Figure 1, Y-axis represents the overall signal value of SEs and typical enhancers (Figure 1).

**FIGURE 2:**
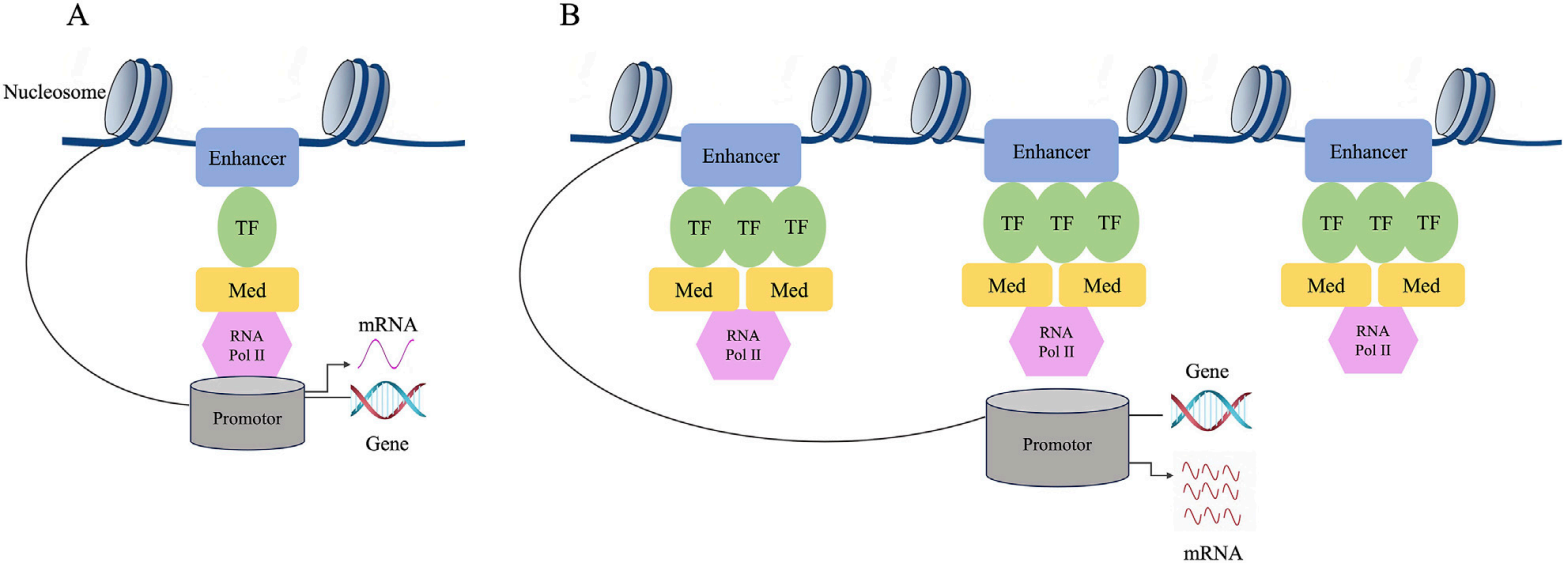
Difference between typical enhancers and SEs. **(A)** The composition structure of enhancers. **(B)** The composition structure of super enhancers. The expression level of genes regulated by SE is much higher than that of genes regulated by typical enhancer. The individual enhancers that make up SEs can activate gene transcription. Compared to typical enhancers, SEs have a stronger transcriptional activation ability, and their associated genes show higher expression levels. SEs may strongly promote the transcription of their target genes.

Based on the large difference in the mean density between SEs and typical enhancers, SEs have a strong transcriptional activation ability, and the expression levels of the genes associated with them are also relatively high. The transcription factors bound by SEs and the chromosome markers associated with transcriptional activity are much higher than those of typical enhancers. As a result, SEs may strongly promote the transcription of their target genes (Whyte et al., 2013) (Figure 2).

## 2 Major characteristics of SEs

### 2.1 Structural features of SEs

**FIGURE 3:**
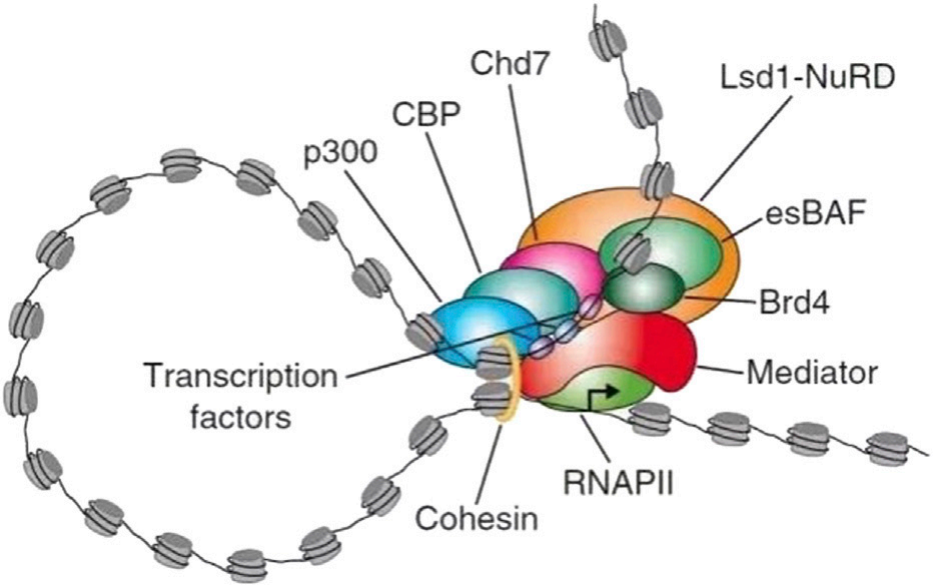
Example diagram of super-enhancer substructure. SEs are a large cluster of transcriptionally active enhancers enriched with a high density of master transcription factors, cofactors, and histone modification marks. Large numbers of transcription factors are required before RNA PII binds to the promoter upstream of the gene and begins transcription. SEs has many TFs binding sites, which can recruit mediators to change the spatial structure of chromatin and promote the interaction of TFs with enhancers, promoters or RNA PII. Recruitment of RNA PII at enhancer precedes loading of RNA PII at promoter of target gene, suggesting that enhancer transcription may regulate recruitment of RNA PII at promoter. In terms of function, SEs can drive the expression of genes that control cell identity and explain cell-type-specific expression patterns in developmental biology, cancer, and other diseases. It has great potential for application in the study of pathogenesis of disease. RNA PII, RNA polymerase II. LSD1-NuRD, Lysine-specific demethylase 1 (LSD1)/nucleosome remodeling and histone deacetylase (NuRD) complex. esBAF, Embryonic stem cell-specific Brahma-associated factor. Bromodomain containing 4 (BRD4, binding to the histone acetylation modification site). CBP, cyclic-AMP response binding protein. CHD7, Chromodomain helicase DNA-binding protein 7.

Most SEs are located in super-enhancer domains (SDs). The eukaryotic genome is a highly ordered, hierarchical structure in which DNA and histones are assembled into nucleosomes, which comprise the chromatin primary structure. Nucleosomes then fold into topologically associating domains (TADs), which are the basic units of chromatin folding and function, and these TADs then further form more complex chromosome structures (Sexton and Cavalli, 2015; Ulianov et al., 2016). TADs are defined by the frequency of DNA–DNA interactions: within TADs, DNA-DNA interactions occur at very high frequencies, and the boundary between each TAD is the region where DNA-DNA interactions are much less frequent (Naumova et al., 2013). Within one TAD, a cohesin can mediate the formation of various secondary structures of gene rings, such as cohesin-associated enhancer-promoter loops and cohesin-associated CTCF loops, which further regulate gene expression (Agrawal and Rao, 2021; Sexton and Cavalli, 2015). Dowen et al. has found that most SEs and their associated genes are in the large CTCF-CTCF loops, approximately 84%, in contrast to only 48% of typical enhancers (Dowen et al., 2014). A SD usually contains a SE that forms a transcription loop along with the genes in the SD to limit activation of the gene transcription. The loss of this constraint may cause the inappropriate activation of neighboring genes and may even be sufficient to result in tumor development because of the mistargeted gene activation (Hu et al., 2023). SEs affect transcription activity by recruiting a high density of master transcription factors and cofactors. Enhancer-associated epigenetic modifications, including Histone H3 lysine 4 monomethylation (H3K4me1) and Histone H3 acetylated lysine 27 (H3K27ac) along with protein 300 (P300), are highly localized, which may drive the expression of cell identity genes and can be used to explain the specific expression pattern of cells (Pott and Lieb, 2015). The SE domain is characterized by a large DNA size, composed of multiple enhancer units, high-density binding of transcription factors and co-factors, open chromatin structure and specific histone modifications. SEs form transcriptional condensates through phase separation, which are cell type-specific and disease-related (Figure 3). These characteristics enable SEs to play a key role in gene expression regulation and make them an important subject for studying cell fate, development, and disease mechanisms. (Hah et al., 2015; Hnisz et al., 2013; Lovén et al., 2013; Xiao et al., 2021). In developmental biology, cancer, and many other diseases, SEs have shown great potential for application in studying etiopathogenesis and effective treatment (Wang et al., 2023).

### 2.2 SEs enrich high density transcription factors, cofactors, and enhancers

Compared to typical enhancers, SEs are large regulatory elements that enable cell type-specific gene regulation to extend over longer regions on DNA (Whyte et al., 2013). The median sequence length of the 231 SEs identified from mouse embryonic stem cells was 8, 876 bp, compared with just 703 bp for the 8, 563 typical enhancers. Similarly, 395 SEs were identified using the key transcription factor PU.1 of mouse pro-B cells, with a median length of 16,800 bp by using ChIP-seq technique, showing a significant difference compared to 13,419 typical enhancers (the median is 490 bp),indicating that SEs acts as a ‘platform’ that brings together developmental internal and external environmental signaling pathways to control temporal and spatial expression of genes (Hnisz et al., 2013).

Studies have found that in the SEs of mouse embryonic stem cells (ESCs), the binding sites of terminal transcription factors such as TCF3, STAT3, and SMAD3 in Wnt, TGF-β, and LIF signaling pathways are similar to binding site maps of the master transcription factors, Oct4, Sox2, and Nanog (Glinsky, 2018; King et al., 2016; Zhang J. et al., 2022). These transcription factors, including Oct4, Sox2, and Nanog, can form complexes with co-activators of the mediator and then bind to enhancers (Kagey et al., 2010). The mediator complex facilitates the ability of enhancer-bound transcription factors to recruit RNA Pol II to the target genes’ promoters (Blayney et al., 2023). Interest in further studies have confirmed that regions with high levels of ESC transcription factors (such as Oct4, Sox2, Nanog, Klf4 and Esrrb) have higher transcriptional activity than typical enhancers and are abnormally insensitive to mediator levels. SEs were found in a wide variety of differentiated cell types, associated with key cell-type-specific genes, which were known to play prominent roles in control gene expression. SEs enrich high density transcription factors, cofactors, and enhancers in order to drive genes which are essential for cell identity in different mammalian cell types (Zamudio et al., 2019). With adjacent enhanced startup unit analysis and dye color transfer conformation capture technology, researchers found that many luxury genes that determine cell fate are regulated by SEs, but most housekeeping genes do not interact with SEs (Whyte et al., 2013). After ESCs were treated with SEs-bound transcription factor inhibitors, it was found that SEs-related genes were preferentially inhibited, and the degree of inhibition was significantly stronger than that of typical enhancer related genes, suggesting that SEs activation is an important event in the expression of key transcription factors in the process of cell fate determination (Whyte et al., 2013; Zamudio et al., 2019).

## 3 Functional characteristics of SEs

### 3.1 Expression and function of eRNAs transcribed from SEs

Noncoding RNAs expressed from enhancers, also named eRNAs, synthesized in the SE region before the transcription of the target gene, with an average length of 350 nucleotides (Andersson et al., 2014). eRNA is classified into two types based on its length, transcription directionality, and polyadenylation status: 1D eRNA and 2D eRNA. Unidirectional transcription generates long (over 4 kb) and polyadenylated eRNA, which is called 1D eRNA (Koch et al., 2011). Bidirectional transcripts generate short (0.5–2 kb) non-adenylated eRNA, which is called 2D eRNA (Kim et al., 2010; Lam et al., 2014). The majority of the eRNAs expressed in human cell types are classified as 2D eRNAs. Normally, the quantity of eRNAs in SEs is 24.3 times that of typical enhancers. In certain macrophages, eRNAs are proved to be expressed in almost all SEs (Hah et al., 2015).

eRNAs can promote the transcription of associated genes and are a genome-wide feature of the functionally active enhancers (Kim et al., 2010). An active enhancer synthesizes eRNA via the direct recruitment of RNA polymerase II through one-way or two-way transcription (De Santa et al., 2010). The transcription of the enhancer and the synthesis of eRNA contribute to the function of the enhancer, and its transcription level is highly correlated with the activity of the enhancer (Lam et al., 2014). Recent studies of eRNAs have been conducted in a variety of cell types, including neurons (Kim et al., 2010), macrophages (Kaikkonen et al., 2013), T cells (Koch et al., 2011), and cancer cells (Hah et al., 2011). In addition, Hah et al. used TLR4 (Toll-like receptor 4) signaling in macrophages as a pattern system to study the role of an SE and its associated eRNA in the inflammatory response (Hah et al., 2015). Studies have shown that the transcription of SE-associated eRNAs is dynamically regulated, and in response to cellular signals, most of the eRNAs in the SEs, which drive the key genes for innate immunity and the inflammatory response, are induced to be transcribed. Surprisingly, the TLR4 signaling suppressor gene is also associated with the SD, along with eRNA transcription suppression. Therefore, both the activation and inhibition of gene expression are regulated by the transcriptional changes of eRNA, and the transcriptional activity of eRNA within the SE may be a key component to understanding the dynamic gene regulatory network (Hah et al., 2015; Tu et al., 2021; Wang et al., 2011).

CRISPR is a highly effective, rapid, and inexpensive gene editing technology, which is usually applied to achieve gene knockout in cells. Since CRISPR-Cas9 was first used as a genome editing tool, its application scope has been continuously expanding. It can not only modify the genomic sequences of cells and organisms but also introduce epigenetic and transcriptional modifications (Moon et al., 2019). Currently, CRISPR-Cas9 technology can be used to identify endogenous enhancer elements and study the impact of their presence or absence on gene expression. eRNAs and the target genes regulated by enhancers are positively correlated at the expression level. Therefore, potential target genes regulated by enhancers can be screened through RNA-level association analysis, and active enhancers in specific cell types can be identified by searching for regions enriched with H3K4me1 and H3K27ac on the genome using ChIP-seq. In terms of elucidating the mechanism of action between enhancers and their regulated target genes, CRISPR-Cas9 technology can be utilized by designing sgRNAs to knockout enhancers, and then conducting subsequent studies on the regulatory mechanism (Ghorbani et al., 2021; Li et al., 2020).

### 3.2 SEs possess greater abilities of transcriptional activation and sensitivity

SEs have shown a strong transcriptional activation ability, and the related genes exhibit a relatively high expression level. Whyte et al. found that unimodal fragments of SEs could generate 3.8 times more luciferase activity than typical enhancers after unimodal clips of 600–1,400 bp in the SEs of mouse ESCs, suggesting that SEs possess a stronger ability to drive the transcription of target genes (Whyte et al., 2013). At the same time, these individual enhancers, which can make up SEs, have shown no superposition or synergistic effect on increasing the regulatory function of gene expression, indicating that one component has a more complex effect on one another’s activity. Some have positive effects on transcriptional activity, while others have negative ones (Hnisz et al., 2015).

SEs have key transcription-factor-dependent characteristics and exhibit cell-type-specific functions, resulting in stronger responses to interference. Silencing the key transcription factor October 4 in mouse ESCs could result in the loss of the pluripotent state. In this process, compared with typical enhancer-associated genes, the expression level of SE-associated genes could be reduced significantly, indicating that SEs have higher sensitivity to the transfection (Ong and Corces, 2011). Transcription factors need to bind to the motifs of genes and then regulate their expression. After analyzing known transcription factors’ binding motifs, Studies have confirmed that SEs are enriched in binding sites for transcription factors that can specify cells or signaling pathways that impinge on them; thus, SEs act as a platform to integrate the environmental and developmental cues necessary to orchestrate spatiotemporally controlled gene expression (Hnisz et al., 2015; Sengupta and George, 2017). Furthermore, SEs are also enriched in signaling pathway transcription factor motifs, which are mainly involved in the binding of terminal transcription factors in response to signaling pathways.

### 3.3 SEs define cellular identity

**FIGURE 4:**
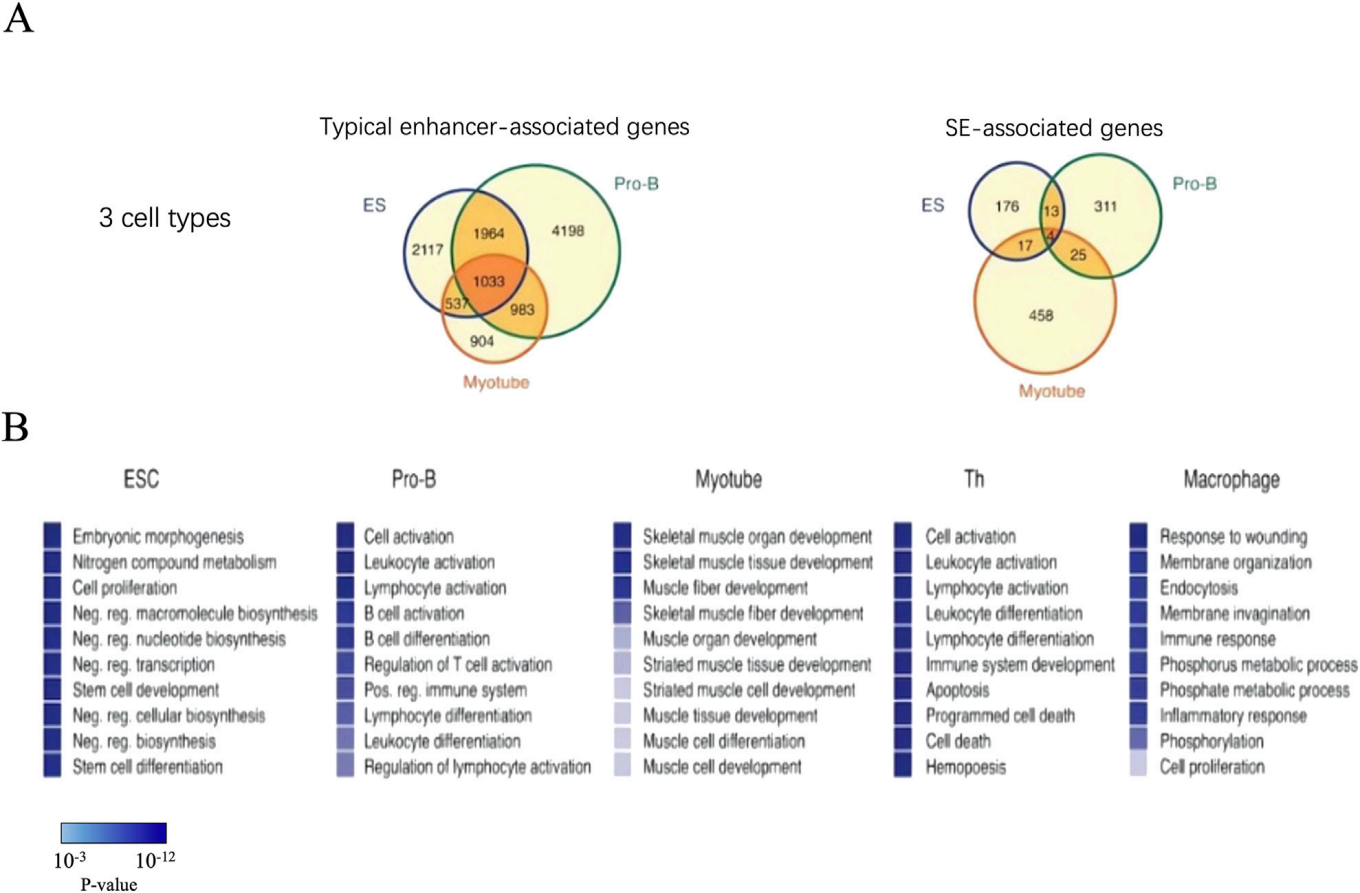
The genes associated with SEs are highly cell type specific. **(A)** There are much more associated genes in the typical enhancers compared with SEs. **(B)** GO analysis in genes associated with SE. The top ten biological process terms were remarkably descriptive of cells’ specific function. P-values corresponding to each of the gene ontology terms are displayed as a color bar, with color scale bar denoted in the figure.

SEs can be identified in any cell types to define the characteristics of cellular identity (Lovén et al., 2013). After analyzing the characteristics of SEs in mouse ESCs, Whyte further explored whether there were similar characteristics in other cell types (Whyte et al., 2013). By using ChIP-seq to analyze mouse pro-B cells, myotubule cells, T cells, and macrophages through the key transcription factors PU.1, MyoD, T-bet, and C/EBPα, respectively, researchers have confirmed the presence of SEs in these cell types. At the same time, these SEs have similar structural characteristics to those identified in the mouse ESCs and are associated with key genes specific to cell types (Di Giorgio et al., 2023; Whyte et al., 2013; Zhang et al., 2008) (Figure 4).

Although SEs can be defined by the certain binding sites of key transcription factors, however, the key transcription factors whether can be used for defining SEs for cell types are not clear. Hnisz et al. explored the use of various enhancer substitution markers, including histone H3K27ac modification, histone H3K4me1 modification, DNase I hypersensitivity sites, and P300, to identify the effect of SEs in ESCs. Therefore, ChIP-seq data from cells of different tissues of 86 individuals were subsequently analyzed using histone H3K27ac modification. The signal intensity of H3K27ac predicted the distribution of the SEs in these specific cell types, which further confirmed that most of the SEs exhibited cell type specificity and defined cell identity characteristics (Lovén et al., 2013). Consistent with these findings, SEs are enriched in binding motifs that correspond to key cell-specific transcription factors compared to typical enhancers. Therefore, histone H3K27ac modification can be used to predict the SEs for specific cell types (Pott and Lieb, 2015).

### 3.4 SE-associated genes specifically respond to signal input

SEs contain DNA motifs of signaling pathway transcription factors, which may bind and respond to the terminal transcription factors of the signaling pathway (Hnisz et al., 2015). In mouse ESCs, SEs are capable of binding to the terminal transcription factors in the Wnt, TGF-β, and LIF signaling pathways more frequently than typical enhancers because of their greater sensitivity to a variety of signals. *In vivo*, after the stimulation or interference of the Wnt, TGF-β, and LIF signaling pathways and then the use of Gene Set Enrichment Analysis (GSEA), scientists found that the expression of SE-associated genes showed greater changes, while the expression levels of typical enhancer-associated genes were much more moderate (Hnisz et al., 2015). The strongly responsive genes associated with SEs mainly include previously reported target genes for these signaling pathways, such as Wnt, TGF-β, and LIF signaling pathways, which play a key role in maintaining the self-renewal and pluripotency of embryonic stem cells. Thus, SEs at least provided an avenue for these signaling pathways through delivering maintenance or modification genes, which partly explains why tumor cells are inclined to form SEs in key tumorigenesis (Sengupta and George, 2017). In summary, SEs can provide a signaling pathway to regulate the expression of cellular identity genes during disease development and progression.

### 3.5 SEs drive the expression of key oncogenes

Many key oncogenes in tumor cells are driven by SEs. Compared with normal cells, tumor cells can build SEs at the sites of oncogenes to drive gene expression during tumorigenesis (Lovén et al., 2013; Pott and Lieb, 2015). c-myc is an important oncogene, and a massive SE has been found in the gene desert of c-myc that cannot be detected in the corresponding healthy cells (Tang et al., 2022). Each type of tumor cell has its own unique or partly overlapping SE site in the genome (Lovén et al., 2013). This functional feature of SEs in cancer cells may be used to identify key oncogenes for the development of targeted drugs (Chipumuro et al., 2014; Trabucco et al., 2015). Cancer cells can construct SEs that drive oncogenes through genetic mechanisms such as mutations, the chromosomal translocations of regular gene SEs, and the local amplification and overexpression of oncogenic transcription factors (Gröschel et al., 2014; Mansour et al., 2014). For example, one subtype of T cell acute lymphoblastic leukemia (T-ALL) arises from somatic mutations at noncoding sites upstream of the TAL1 gene in which a binding site of transcription factor MYB is imported, thus forming an SE and, finally, resulting in the overexpression of TAL1 (Mansour et al., 2014).

In tumor cells, signaling pathways regulate the activity of SEs in multiple ways. Licht J.D., a professor at Northwestern University, and colleagues found that the activity of Ras-Erk is closely related to the activity of SEs: the inhibition of Ras protein activity results in the disappearance of SE-related features (such as H3K27ac), decreased activity, and the further decreased transcription of related genes. Activating Ras can enhance the SE activity of oncogenes (Nabet et al., 2015). On the other hand, pro-cancer signaling pathways can modulate the activity of SEs by manipulating transcription process. The transcriptional pausing means a state in which the active RNA Pol Ⅱ stops the transcription near the promoter (Adelman and Lis, 2012). However, in Hippo signaling pathways in liver cancer cells, the absence of such inhibition can cause YAP (Yes-associated protein) to move into the nucleus and bind to the SE before recruiting Mediator complexes and Cyclin-dependent kinase 9, inducing the release of the suspended RNA Pol II into the extension state and promoting gene transcription (Galli et al., 2015; Yimlamai et al., 2015). Therefore, in liver cancer, YAP promotes oncogene transcription by activating SEs. Compared with normal lung tissue, the H3K27ac profile of LUAD cell lines showed cancer-specific and normal-specific SEs (Tang et al., 2022). Cancer-specific SE target genes were enriched in LUAD driver genes and tumor signaling pathways, while normal-specific SE target genes were associated with immunity (Xu et al., 2024). The HOXB gene cluster locus is one of the most common SE-associated elements (Ying et al., 2020). The HOXB cluster-associated SE is detected in primary CRC tissues but not normal colon tissues, suggesting it is a specific element to CRC. HOXB8 overexpression is essential for maintaining the malignant phenotype of CRC and is regulated by SEs associated with the HOXB cluster (Huang et al., 2021).

These studies suggest that SEs can be used as channels to link oncogene signaling pathways and maintain gene transcription expression in tumor cells. However, further studies have found that the regulation of SEs by signaling pathways is related to the dynamic binding of transcription factors in the SE region. For example, in leukemia cells caused by an abnormality in NOTCH1, NOTCH1 is generally bound to the genome, but only 10% of NOTCH binding sites respond to upstream signaling, and the majority of these 10% binding sites reside in SEs (Wang et al., 2014).

## 4 Transcriptional regulation by SEs in immune responses within the tumor microenvironment

In tumor cells, oncogenes are transcribed and activated, mediating cell proliferation and immortalization (Hanahan and Weinberg, 2011). Therefore, the inhibition of oncogene transcription is a potential therapeutic target. However, this target faces a major challenge: transcription is the most basic function of cells, and the transcriptional inhibition of oncogenes may lead to a broad-spectrum inhibition of cell gene transcription (Sengupta and George, 2017; Sur and Taipale, 2016). Nowadays, clinically used transcriptional inhibitors should specifically inhibit oncogenes and have little effect on normal cell transcription (Didiasova et al., 2018). Researchers have found that there are a large number of “tumor-specific transcripts” in triple-negative breast cancer cells, and the world’s first “tumor-specific transcript” map of triple-negative breast cancer has been successfully drawn. Further research has discovered that SEs can activate the expression of “tumor-specific transcript” MARCO-TST in triple-negative breast cancer, and for the first time confirmed that BET inhibitors can be a potential treatment option for patients with triple-negative breast cancer (Yang et al., 2022). Compared with individual oncogenes or molecules, the maintenance of tumor cell characteristics is more dependent on the activity of SEs. This implies that SEs are more ideal targets for anti-cancer treatment. Therefore, targeting and inhibiting the activity of SEs or knocking out SEs fragments may become a new approach for tumor treatment. However, as transcription is a biological process that occurs universally in the body, it cannot be inhibited as a whole. Highly specific inhibition is required for anti-tumor treatment. Therefore, it is necessary to search for a possible intervention target among the small molecules involved in the SEs mechanism.

Transcriptional initiation, suspension, elongation, and other processes are regulated by transcription factors. Studies have shown evidence that the SE regulation of transcription relies on BRD4, mediator complexes, cell-cycle-dependent kinase 7 (CDK7) complexes, and the CDK9 transcription complex (Adelman and Lis, 2012). In addition, BRD4 promotes the assembly of SEs by recruiting the mediator complex, thereby promoting the release of suspended RNA Pol II (Di Micco et al., 2014). CDK12/13 can accelerate RNA Pol II transcription. Therefore, it is generally believed that the mediator complex, BRD4, and CDK, the key regulatory factors of the SE regulation of transcription, are conducive to the development of new tumor therapeutic targets. Based on the key nodes of transcriptional inhibition mentioned above, the main drug types currently available for the targeting of SEs are ① inhibitors or decomposers of BRD family proteins; ② CDK7 inhibitor; and ③ other types of inhibitors (Bartkowiak et al., 2010; Liang et al., 2015).

JQ1 inhibits the interaction between BRD4 and acetylated proteins by binding to a domain of BRD4 (Delmore et al., 2011; Filippakopoulos et al., 2010), which limits the binding of BRD4 to the H3K27ac site of the SE and inhibits the interaction between the SE and the promoter, thereby affecting oncogenes’ transcription (Lovén et al., 2013). Since the transcription regulated by SEs is particularly sensitive to changes in the concentration of transcription factors, JQ1 treatment can preferentially prevent the binding of BRD4 to the acetylation modification sites on SEs, thus specifically inhibiting the transcriptional activation mediated by SEs (Lovén et al., 2013; Shin, 2018). In addition, the BRD inhibitors iBET762, OTX015, CPI0610, and iBET151 were also found, and the first three have entered clinical trials (Amorim et al., 2016). dBET series compounds are more specific BRD4 inhibitors developed based on the chemical structure of JQ1, which can specifically mediate the degradation of BRD family proteins, thereby preventing these proteins from recognizing the acetylation sites of SEs, affecting the activity of the SEs, and inhibiting transcription (Qian et al., 2023; Tasdemir et al., 2016). Studies have shown that BETd-246 can target the specific degradation of BRD family proteins, and it also shows a better therapeutic effect in triple-negative breast cancer than iBET-211 (Bai et al., 2017).

THZ1 is a CDK7-specific inhibitor to which some SE-mediated tumor cells are highly sensitive (Kwiatkowski et al., 2014). THZ1 can covalently bind to CDK7 cysteine 312, inhibiting CDK7 kinase activity and the phosphorylation of CDK7 for Pol II CTD’s fifth serine, thereby blocking transcription initiation and reducing the amount of promoter-proximal paused RNA Pol II. The SE of the main control suspension RNA Pol II is released. Then THZ1 processes RNA Pol at the bottom of the promoter II, reduces the enhancer of the RNA Pol II combination, and finally suppresses transcription (Zhang et al., 2020). After THZ1 treatment, the SE activity decreased, which leads to the transcriptional inhibition of a variety of oncogenes, thus inhibiting the growth and proliferation of a variety of tumor cells. Syros developed SY-1365 as a specific inhibitor of CDK7 that selectively inhibits a variety of solid tumors (breast, ovarian, and small-cell lung cancers) and blood cancers (acute myeloid leukemia and acute lymphoblastic leukemia).

CDK12 is a kinase that regulates transcription elongation. In T cell leukemia, THZ531 can specifically inhibit CDK12/13 and effectively inhibit super-enhancer-mediated gene expression (Zhang et al., 2016). Inhibiting the activity of mediator kinase (CDK8/19) in acute myeloid leukemia can upregulate the SE activity related to a tumor suppressor and activate the expression of the tumor suppressor gene, thus achieving anti-leukemia activity (Pelish et al., 2015). Similarly, the CDK 4/6 inhibitor LEE011 selectively inhibits CDK4, downregulates cyclin D1-related SE activity, and effectively promotes the apoptosis of Ewing’s sarcoma cells (Kennedy et al., 2015).

GZ17-6.02 can affect gene acetylation, reduce the transcription of master transcription factors, proteins in the sonic hedgehog protein pathway, and stem cell markers (Ghosh et al., 2019). In pancreatic ductal adenocarcinoma, GZ17-6.02 can decrease the occupancy of master transcription factor Oct4 in SE regions, reduce the activity of SEs, and thereby inhibit the growth of pancreatic ductal adenocarcinoma cells (Ghosh et al., 2019). GZ17-6.02 can also inhibit the growth of malignant glioblastoma by down regulating the expression of SE genes (Choi et al., 2022). Additionally, Syros Pharmaceuticals has utilized its gene control platform to identify acute myeloid leukemia patients and subgroups of myelodysplastic syndrome with SEs of RARA or IRF8 genes and discovered biomarkers that can recognize these SEs. These SEs are believed to drive the overexpression of RARA or IRF8 genes, leading to tumor development by targeting immature, undifferentiated, and proliferating cells. The retinoic acid receptor-α agonist SY-1425 can promote the differentiation of acute myeloid leukemia cells with high expression of RARA or IRF8 genes, thereby inhibiting tumor growth (McKeown et al., 2019).

SEs have become a highly controversial topic in both clinical and basic research, with their functions and potential clinical therapeutic prospects drawing significant attention. Currently, a large number of BET family protein inhibitors or degraders and CDKs inhibitors have been included in clinical studies targeting SEs, and their value in anti-tumor treatment is gradually being discovered. However, it is worth noting that targeting SEs for cancer treatment may cause significant adverse reactions, as some normal genes will also be inhibited when SEs are blocked. For instance, studies have shown that THZ1 can inhibit myogenic differentiation, indicating that THZ1 may cause adverse reactions to muscle function during the treatment process (Dutta et al., 2023). Additionally, interfering with the generation of oncogenic SEs through the CRISPR/Cas9 gene editing system can block SEs, regulate SEs-driven oncogenes, and prevent tumor occurrence. It is also possible to exert anti-tumor effects by knocking out oncogenic SEs through the CRISPR/Cas9 gene editing system. However, due to the off-target effects of the CRISPR/Cas9 gene editing system, any non-target site gene editing is associated with clinical risks (Zheng et al., 2020). ChIP-seq is one of the main techniques for identifying SEs, but its application in SE research still has several limitations. For instance, the ChIP-seq experiment involves multiple steps, including cross-linking, chromatin fragmentation, immunoprecipitation, library construction, and sequencing, and each step may introduce errors (Anzawa et al., 2020). The uniformity of chromatin fragmentation, the quality of antibodies (such as the specificity and affinity of H3K27ac antibodies), and the efficiency of immunoprecipitation all affect data quality (Anzawa et al., 2020). Moreover, traditional SE identification methods (such as the ROSE algorithm) mainly rely on the signal intensity of H3K27ac ChIP-seq, but this method cannot directly verify whether SEs truly regulate gene expression, resulting in a high false positive rate (Mack et al., 2018). Research has found that in colorectal cancer, only 16.1% of ROSE-predicted SEs are significantly correlated with gene expression, indicating that most predicted SEs may not be functional. Currently, emerging technologies such as CUT&RUN and CUT&Tag, which have lower cell requirements, higher throughput, and simpler operation procedures, are gradually replacing ChIP-seq in chromatin mapping studies (Shinkai et al., 2025). These technologies can more efficiently capture the binding sites of transcription factors and histone modifications, but they have not yet been fully standardized for SE research. In summary, although ChIP-seq still holds an important position in SE research, its limitations have prompted scientists to explore more efficient and accurate experimental and computational methods.

## 5 SEs harbor disease-susceptibility SNPs across multiple conditions

It is currently known that there are over 80 types of autoimmune diseases, which affect 3%–5% of the total population in the United States (Jacobson et al., 1997; Marrack et al., 2001). Scientists have found that human leukocyte antigens (HLAs) can be divided into two classes: class I and class II. HLA class I is expressed in almost all cell types and presents peptide antigens on the cell surface. In contrast, HLA class II is significantly expressed only on dendritic cells and B cells and presents antigens on the surface of both types of cells. Single-nucleotide polymorphisms (SNPs) in the HLA class I and class II genes can encode on chromosome 6p21.3 and are associated with various autoimmune diseases (Okada et al., 2014; Vahedi et al., 2015). Moreover, disease-related mutations can be enriched in SEs. The results suggest that the distribution of this variation is disproportionate and that for most disease types, disease-related SNPs are enriched in the SEs found in disease-related cell types (López-Isac et al., 2019). This correlation also explains the role of SE-associated genes in defining cellular identity, and changes in the expression of identity genes may lead to the occurrence of related diseases. For example, of the 27 SNPs known to be associated with Alzheimer’s disease, approximately 19% (5/27) occur in SEs in brain tissue (Chapuis et al., 2013; Dunn et al., 2025). A similar situation has been observed for a variety of immune-related diseases including type I diabetes (Cooper et al., 2008), systemic lupus erythematosus (Almlöf et al., 2017; Zhang Y. et al., 2022), rheumatoid arthritis (Jadhav et al., 2022; Okada et al., 2014; Tsuchiya et al., 2021), multiple sclerosis (Sawcer et al., 2011), primary biliary cirrhosis (Cavalli et al., 2016), Crohn’s disease (Franke et al., 2010), Asthma (Ferreira et al., 2011), and atrial fibrillation (Smith et al., 2012), which exhibit an enrichment of SNP loci in a T-cell-specific SE region (Vahedi et al., 2015). Through studying the lead expression-modulating SNP, scientists also uncovered an NF-κB-driven regulatory circuit which constrains T-cell activation via the dynamic formation of a SE that upregulates TNFAIP3 (Bourges et al., 2020). Therefore, SEs can be used to study the pathogenesis of these diseases, while drugs that affect the genes associated with the specific SEs may be more effective in clinical applications. At the same time, the combination of cell-type-specific SEs with human genetic information also provides a scientific basis for the development of targeted drugs.

As we previously mentioned, rheumatoid arthritis (RA) is a systemic autoimmune disease characterized by chronic synovial inflammation and progressive joint destruction (Odegård et al., 2005; Yelin, 2007). Proinflammatory cytokines such as tumor necrosis factor α (TNF-α), interleukin 1β (IL-1β), and proteases (e.g., MMP9 and MMP3) increase significantly in the synovial fluid (McInnes and Schett, 2011; Redlich and Smolen, 2012). Thus, TNF inhibitors, interleukin receptor inhibitors, and other small-molecular-weight compounds such as Janus kinase (JAK) inhibitors have been frequently and actively applied in clinical practice (Kubo et al., 2014; Singh et al., 2019; Tanaka, 2019). However, some patients relapsed or showed no remission, indicating that the existing drugs had little effect on them. As discussed previously, some SNPs are known to be associated with high susceptibility to RA. In addition, the results of GWAS analysis have revealed that approximately 101 SNPs are connected to RA and cover almost 50% of the genomic variants underlying the susceptibility to RA (Peeters et al., 2015; Viatte et al., 2013). Compared with typical enhancers, SEs harbor 3.2 times more SNPs that are associated with susceptibility to RA, indicating that the SNPs related to RA can strongly take part in SE-mediated transcriptional regulation (Teumer et al., 2018) and may serve as a new avenue for RA therapy.

The basic leucine zipper transcription factor 2 (BACH2) protein is an important transcription factor for the maintenance of immune homeostasis by Treg cells (Vahedi et al., 2015). In T cells, BACH2 inhibits the expression of genes encoding various cytokines, including IFN-gamma, and cytokine receptors. Gene mutations at the BACH2 locus are associated with RA. The knockdown of the BACH2 gene induces the expression of various cytokines and their receptors (Vahedi et al., 2015). Although the BACH2 protein negatively regulates the expression of eRNAs, the gene itself is uniquely regulated by SEs (Vahedi et al., 2015). However, it remains unclear which eRNA induces the expression of the BACH2 gene and how it achieves this goal. Tofacitinib, can inhibit JAK1/3, thereby reducing the expression of several genes related to the susceptibility of RA. Studies have confirmed that, compared with genes not regulated by SEs, its inhibitory effect on the expression of genes regulated by SEs is more significant. (Vahedi et al., 2015). This suggests that JAK/STAT signaling factors can regulate the expression of RA susceptibility-related genes through SEs.

Systemic lupus erythematosus (SLE) is an autoimmune disease that follows a chronic course or repeated cycles of remission and relapse and predominantly affects women. According to GWAS, approximately 60 disease-susceptibility SNPs have been identified in European SLE patients (Teruel and Alarcón-Riquelme, 2016), while nine new disease-susceptibility loci were identified in Chinese patients (Han et al., 2009). Researchers have discovered that A Disintegrin And Metalloproteinase (ADAM)-like decysin-1 (ADAMDEC1) are important for proteolytic cleavage (Shi et al., 2018). ADAMDEC1 is closely related to ADAM28, which plays a key role in maintaining the acute inflammatory process. What’s more important is ADAMDEC1 is overexpressed in monocytes of SLE patients, and its expression can be induced by pro-inflammatory cytokine stimulation. (Guo et al., 2022). Moreover, inflammatory stimulation leads to the recruitment of NF-κB and P300 upstream of the ADAMDEC1 gene. In the absence of such combination, the induction of ADAMDEC1 expression was inhibited (Shi et al., 2018). After enhancing the activity of P300 marked with H3K27ac, eRNA-157 promoted the induction of ADAMDEC1 gene expression by looping between the promoter and SE. eRNA-157 is a short noncoding RNA in a non-polyadenylated state produced through bidirectional transcription, while ADAMDEC1 mRNA is a long-coding RNA in a polyadenylated state generated via unidirectional transcription. Although eRNA-157 involves the induction of ADAMDEC1 mRNA, whether the latter regulates the former is still unclear (Yamagata et al., 2020).

The programmed cell death 1 gene, PDCD1, encodes a programmed death 1 (PD-1) protein, which is an important immune checkpoint. Mice with PDCD1 knockout exhibit SLE-like pathology (Nishimura et al., 1999). What’s more, SNPs in the PDCD1 gene are also associated with SLE (Prokunina et al., 2002). Importantly, the PDCD1 gene has a SE structure in CD4-naive T cells and may be regulated by SEs (Khan and Zhang, 2016). Due to the different types of simulation, SE function may be impaired, thus potentially promoting the pathogenesis of SLE. As mentioned above, the eRNAs and SEs that may be involved in the control of the pathological conditions of SLE patients have been identified. In the future, their functions will be elucidated at an individual level using mice and other disease models.

Multiple sclerosis (MS) is also a complex autoimmune disease that is caused by a combination of many risk factors, including genetic mutations and vitamin D deficiency. SNPs associated with the susceptibility to MS are observed at and around vitamin D receptor (VDR)-binding sites (Sospedra and Martin, 2005). Lu et al. classified SEs bound to VDR (termed VDR SE, VSE) into three types VSE1, VSE2 and VSE3. Several SNPs associated with MS susceptibility were detected in the VSE domain, particularly in the VSE3 subdomain (Lu et al., 2019). Based on these findings, disease-susceptibility SNPs within SEs are assumed to regulate SEs. However, it remains unclear how the presence of SNPs associated with MS susceptibility affects the expression of eRNA through the interaction of VDR that binds to vitamin D and chromatin, which seems to be an issue for further investigation.

In the case of inflammatory bowel disease (IBD), it has been found that approximately half of the risk SNPs are located in the SE regions of CD4 T cell activation (Vahedi et al., 2015). Graves’ disease (GD) is associated with excessive humoral immunity, due to the production of autoantibodies against the thyroid-stimulating hormone (TSH) receptor 1 (Klecha et al., 2008). GWAS has identified 101 SNPs associated with susceptibility to GD (Teumer et al., 2018). At the same time, atopic dermatitis (AD) causes chronic and recurrent inflammatory allergic reactions, such as skin itching and peeling. In the future, it will be of great significance to clarify the role of SE in various autoimmune diseases, including the aforementioned ones (Jin et al., 2016). Whyte et al. reported that SEs are *cis*-regulatory elements and form loops with promoters (Whyte et al., 2013). In addition, individual SEs form loops with many promoters, thereby regulating the expression of many target gene clusters (Cong et al., 2019). Therefore, an important theme for future research might be to use GWAS analysis to identify disease-specific SNPs for each autoimmune disease, to identify SNPs important for chromatin interactions through linkage analysis, and to measure actual interactions using chromosome conformation capture analysis.

Although SEs serve as key elements in gene regulation, the SNPs related to them have a significant association with disease mechanisms, there still existed the potential contradictions about the roles of SEs in diseases. Studies have verified the same SE-SNP may have opposite effects in different tissues. For instance, rs12740374 (which regulates SORT1 expression) reduces blood lipids in the liver but may promote atherosclerosis in the vascular wall (Al-Eitan et al., 2020). SE often regulates multiple genes, while SNPs may only affect one of the targets. For instance, rs4791078 is located in a SE involved in heart development, but the gene network it regulates through SMAD3 remains incompletely defined (Nasser et al., 2021). In cancers, SE can both activate tumor suppressor genes (such as TP53) and drive oncogenes (such as MYC), depending on the cellular context (Chen et al., 2024; Kubota et al., 2019). Current functional prediction tools (such as DeepSEA) have limited accuracy in annotating non-coding variants, and experimental validation (such as MPRA) is costly, which may lead to false positives/negatives. In conclusion, SE-related SNPs directly participate in disease occurrence by regulating the expression of key genes. However, tissue specificity, functional redundancy, and technical limitations lead to contradictions in their roles. In the future, it is necessary to combine high-throughput functional experiments with clinical data to elucidate the mechanism of SE more accurately in diseases.

## 6 Discussion

A SE is defined as a large cluster of transcription enhancers that can drive cell-identity-defining genes expression. SEs exhibit unique structural and functional properties compared with typical enhancers; however, at present, there is still a lack of clear rules to define SEs. The mathematical method to distinguish SEs from typical enhancers is mainly based on the difference in the signal strength of active enhancer markers. Whether SEs can be defined as distinct entities still needs further research and verification (Pott and Lieb, 2015; Whyte et al., 2013). In addition, recent research has allowed for an increasing recognition of the many functional similarities between promoters and enhancers. Enhancers have shown the characteristic of driving gene expression, and some promoters have also displayed the function of strengthening gene expression, making it necessary to reevaluate the traditional definitions of enhancers and promoters (Andersson, 2015; Kim and Shiekhattar, 2015). Nevertheless, SEs have shown their value in areas such as cell identity determination and disease pathology. To further explore SEs and their roles in defining cell identity and participating in disease processes, relatively systematic SE database platforms, such as SEA and dbSUPER, have been established recently, and relevant analysis tools have been integrated that will promote the research and understanding of SEs (Khan and Zhang, 2016; Wei et al., 2016). SEs are enriched in key genes that control cell identity, and the expression of many key cancer tumor cell genes is driven by SEs. The characteristics of common diseases related to the significant variation in SE enrichment can be used to identify the key cell-type-specific transcription factors and determine key oncogenes, and variations in associated loci have shown great application potential. With further research developments, SEs may provide new ideas for the development of treatments for common diseases such as cancer and other conditions.

## Glossary

ADAMDEC1: A Disintegrin And Metalloproteinase (ADAM)-like decysin-1
BACH2: Basic leucine zipper transcription factor 2
BRD4: Bromodomain containing 4
CBP: Cyclic-AMP response binding protein.
CDK7: Cell-cycle-dependent kinase 7
CHD7: Chromodomain helicase DNA-binding protein 7
CNS system: Central nervous system
eRNAs: Enhancer RNAs
esBAF: Embryonic stem cell-specific Brahma-associated factor
ESCs: Embryonic stem cells
GD: Graves’ disease
GSEA: Gene Set Enrichment Analysis
GWAS: Genome-wide association studies
HLAs: Human leukocyte antigens
H3K27ac: Histone H3 acetylated lysine 27
H3K4me1: Histone H3 lysine 4 monomethylation
IBD: Inflammatory bowel disease
IL-1β: Interleukin 1β
JAK: Janus kinase
LSD1-NuRD: Lysine-specific demethylase 1
MS: Multiple sclerosis
NuRD: Nucleosome remodeling and histone deacetylase complex
OSN: Nanog
PDCD1: Programmed cell death 1 gene
PD-1: Programmed death 1
Pro-B: Proliferating B cells
p300: Protein 300
RA: Rheumatoid arthritis
RNAPII: RNA polymerase II
SDs: Super-enhancer domains
SEs: Super-enhancers
SLE: Systemic lupus erythematosus
SNPs: Single-nucleotide polymorphisms
TADs: Topologically associating domains
T-ALL: T cell acute lymphoblastic leukemia
Th: helper T cell
TLR4: Toll-like receptor 4
TNF-α: Tumor necrosis factor α
TSH: Thyroid-stimulating hormone
VDR: Vitamin D receptor
YAP: Yes-associated protein
